# Predicting dental caries increment using salivary biomarkers in a remote Indigenous Australian child population

**DOI:** 10.1186/s12903-021-01702-0

**Published:** 2021-07-23

**Authors:** Surani Fernando, Santosh Tadakamadla, Jeroen Kroon, Ratilal Lalloo, Newell W. Johnson

**Affiliations:** 1 School of Medicine and Dentistry, Griffith University, Brisbane, QLD Australia; 2 Menzies Health Institute Queensland, Griffith University, Brisbane, QLD Australia; 3 School of Dentistry, The University of Queensland, St Lucia, Australia; 4 Faculty of Dentistry, Oral and Craniofacial Sciences, King’s College London, London, UK

**Keywords:** Indigenous children, Dental Caries, Increment, Risk indicators, Caries status, Salivary biomarkers

## Abstract

**Background:**

The burden of childhood dental caries amongst Indigenous Australians is higher than in other Australians. Because of differences in lifestyle and the evolutionary history of the oral microbiota, associated risk indicators may differ. Here, we evaluate associations between caries increment, salivary biomarkers and baseline caries among children aged 5–17 years residing in a remote rural Indigenous community.

**Methods:**

This study was part of a trial assessing cost-effectiveness of an intervention to prevent dental caries among children. Baseline epidemiology and application of topical caries-preventive measures was conducted in 2015, followed-up in 2016 and 2017. Children who did not consent or failed to attend the prevention visits but did attend for follow-up epidemiology constituted a natural comparison group for evaluating the intervention. Saliva flow, pH, buffering and bacterial loads were measured at all visits. Caries was scored by the International Caries Detection and Assessment system. Outcome was caries increment. Explanatory variables were sex, being in experimental or comparison group, baseline caries, saliva flowrate and buffering, pH, and salivary loads of mutans streptococci (MS), Lactobacilli (LB), and yeast. Chi Square tests compared caries incidence in relation to explanatory variables and Generalised Linear Models explored associations between explanatory and outcome variables.

**Results:**

Of 408 participants at baseline, only 208 presented at 2-year follow-up. Of caries-free children at baseline, significantly fewer had incipient (p = 0.01) and advanced (p = 0.04) caries after two years. Children in the experimental group experienced fewer tooth surfaces with advanced caries (p = 0.02) than comparison children. Having caries at baseline (p = 0.02) and low salivary flow-rates (p < 0.001) saw a significant increase in advanced caries after two years. Children with high salivary loads of MS (p = 0.03) and LB (p = 0.004) experienced more advanced carious surfaces. Multivariable analysis revealed 58% reduction (p = 0.001) in advanced caries among children with high salivary flow rates. Caries increment was 61% (p = 0.03) more for incipient and 121% (p = 0.007) more for advanced caries among children who harboured higher loads of MS.

**Conclusion:**

As with other ethnicities, children with low salivary flow and those with high MS had higher incipient and advanced caries increments after two years. Such risk assessments facilitate targeted preventive interventions for such communities.

*Trial registration*: Australian New Zealand Clinical Trials Registry (ANZCTR), No: ACTRN12615000693527: 3 July 2015.

## Background

In 2016, there were an estimated 798,400 Aboriginal and Torres Strait Islander people (hereinafter respectfully referred to as Indigenous Australians) living in Australia, representing 3.3% of the total Australian population [1]. The burden of communicable diseases and chronic health conditions amongst Indigenous Australians is substantially higher than that of other Australians [2], irrespective of age groups and health and well-being indices [3]. Reasons for these differences are multifactorial including socioeconomic disadvantage, poor access to health services, poor nutrition, heavy use of alcohol and smoking and genetic variations [4]. Similarly, Indigenous Australian children experience worse oral health than non-Indigenous children [5]. Regardless of many efforts, health inequalities, including oral health, between Indigenous and non-Indigenous Australians have remained for decades [6]. For example, differences in prevalence of dental caries indicators (26% for decayed teeth, 20% for missing teeth and 9% for filled teeth [1]) between Indigenous and non-Indigenous citizens were larger in Australia compared to Canada and New Zealand [7]. Recognising these historical disparities, the Council of Australian Governments (COAG), committed to ‘close the gap’ in disease levels and in life expectancy between Aboriginal and Torres Strait Islander and non-Indigenous Australians by 2030 [8]. The “Closing the Gap Framework” was initiated by the Australian Federal Government in 2008, aiming to reduce inequalities in life expectancy, children’s mortality, education, employment, health, economic participation, healthy homes, and in governance and leadership of and by Indigenous Australians [9]. Encouragingly, in some parts of the country, there have been significant accomplishments in some key areas of health and education according to the most recent report from Queensland [10].

In the Northern Territory in 2020, it was reported that Indigenous children aged 5–10-years (44%) were more likely to have had at least one deciduous tooth with untreated decay than non-Indigenous children (26%), and among 6-to 14- year old’s, Indigenous children were more likely to have had at least one permanent tooth with untreated decay than non-Indigenous children [11].

It is, therefore, clear that ways to identify and to predict Indigenous children at risk from severe dental caries are needed, so that targeted preventative measures can be undertaken. Saliva acts as the first line of defence, by dilution of toxins and via a wide range of non-specific [innate] and acquired immune mechanisms: it lubricates, assists mastication, swallowing, taste, and digestion of food [12]. As with blood and tissue biopsies, oral fluids are a source of biochemical data carrying prognostic and diagnostic information. It is an easily accessible and a non-invasive alternative to traditional diagnostic sources [13]. Saliva is the main biological factor protecting teeth against demineralization [13] by diluting and buffering acids and provides the calcium and phosphate ions for remineralisation. Caries risk assessment has, therefore, mainly focused on saliva as a diagnostic fluid, in addition to measures of past dental disease [14]. Commonly used biomarkers are concentrations of mutans Streptococci (MS) and lactobacilli (LB) in saliva, saliva flow rate and buffering capacity and, especially, past caries experience as a clinical marker [15–17]. Even though many studies have shown that past caries experience is the strongest predictor of future caries [18], salivary biomarkers are promoted by many, especially the dental industry which sells easy-to-use chairside kits for the purpose [12]. Perhaps the greatest value of these is in monitoring compliance with good oral hygiene and dietary practices [12].

The balance of risk factors, and thus the utility of salivary biomarkers as risk indicators among remote Indigenous communities in Australia [19] may or may not be similar to other non-Indigenous and Indigenous communities around the world [20–22]. Colonisation and trans-generation inequalities have negatively impacted the oral and general health of Indigenous Australians. Therefore, it cannot be assumed that the oral microbiome and its potential for disease and maintenance of oral health is the same as that described in modern Western populations, as the oral microbiota of Indigenous Australians has been adapted over thousands of years of evolution [23, 24]. Hence, the objective of this study was to evaluate associations between dental caries increment and salivary load of bacteria (MS, LB), yeasts, salivary pH, flow rate and buffering capacity among children living in a remote Indigenous community in Far North Queensland. This study is unique in that we were able to measure caries increment among an Indigenous child population after two years. The results will help determine the ability of salivary biomarkers and of past caries experience in predicting future caries in this community. More sophisticated analyses of the oral microbiota are published elsewhere [23].

## Methods

This study was approved by the Griffith University Human Research Ethics Committee (GU Ref No: DOH/05/15/HREC) and the Far North Queensland Human Research Ethics Committee (FNQ HREC/15QCH/39–970). The Department of Education and Training (Queensland Government) also provided formal permission to approach potential research participants in schools. Site Specific Approval was authorised by the Torres and Cape Hospital and Health Service. Signed informed consent was obtained from parents or guardians of all children in the study. Consent for publication is covered by the Ethics Approvals and the Information and Consent Forms signed by Parents or Guardians of the children.

### Study design and participants

This study was part of a trial assessing the effectiveness and cost-effectiveness of a single annual professional intervention for the prevention of childhood dental caries in a remote rural Indigenous community [25]. All school children aged 5–17 years attending the two primary and one secondary school campuses in the Northern Peninsula Area (NPA) of Far North Queensland (FNQ) were invited to participate. Data collection protocol and caries risk assessment processes are detailed in the published protocol paper [25]. There were no inclusion or exclusion criteria: the consent process and compliance to attend appointments drove the eventual number of participants. All consenting children underwent a detailed dental examination and an investigation of salivary biomarkers using commercially available chairside test kits to measure flow rate, pH, buffering capacity and later cultured for bacterial assessment [26]. Dental caries status was assessed by three trained and calibrated examiners using the International Caries Detection and Assessment system (ICDAS-II) [27]. The intervention study involved provision of any necessary restorative treatment, provided by the research team, followed by two annual applications of fissure sealant to appropriate teeth, swabbing the dentition with povidone iodine and application of fluoride varnish to the intervention group. A high proportion of children who were consented to the study did not participate in the preventive intervention. This is likely due to the fact that two consents were required: (1) to be surveyed/examined by the research team epidemiologists at schools, (2) to receive dental treatment and the preventative intervention by our research team clinicians. Even though free transport was provided, the response rate for receiving preventive care was low. Further, because we were interested in examining the programmatic effect of the intervention, participants were not randomly allocated to treatment and control arms. There was only 1 annual application for the intervention group, based on selection criteria, and only where required. Sealants were only given in the second annual appointment if there were newly erupted teeth and if sealants were lost from previously sealed teeth. Usual care was provided for a comparison group, which was generated by children who attended for epidemiological assessments but who declined to receive the preventative treatments: usual care consisted of availability of symptomatic treatment from a public sector dentist available one or two days per week.

The baseline survey was carried out in late 2015 and subsequent follow-up visits were conducted at the same time of year in 2016 and 2017. A total of 600 children were invited to participate and at baseline there were 408 participants of which 292 were compliant with saliva analysis. At the 2-year follow up, a total of 208 children participated in clinical examinations and were used for the current analysis.

### Outcome variable

The outcome of the present analysis is caries increment. The paper-based form was based on ICDAS-II guidelines to record caries. This was defined as the sum of tooth surfaces that had an ICDAS code of 0 (sound tooth surface: no evidence of caries after air drying for 5 s) at baseline and had any ICDAS codes from 1 to 6 at 2-year follow up (1 = first visual change in enamel, opacity or discoloration, white or brown, visible at the entrance to the pit or fissure after prolonged air drying; 2 = distinct visual change in enamel when wet or dry; 3 = localized enamel breakdown without clinical visual signs of dentinal involvement; 4 = underlying dark shadow from dentine; 5 = distinct cavity with visible dentine; 6 = extensive cavity with visible dentine) in both deciduous and permanent dentitions [28]. Caries increment was calculated for each individual child in the sample. This paper assesses explanatory variables in relation to two specific outcome variables: (1) incipient caries—determined as the number of tooth surfaces that had no caries at baseline (ICDAS 0) but showed ICDAS codes of 1 or 2 at the 2-year follow up; and (2) overt caries—defined as the number of tooth surfaces that had no caries at baseline (ICDAS 0) but presented with any of the ICDAS scores 3 to 6 at the 2-year follow up. For bivariate analysis, caries incidence was used as the outcome, which is defined as the number of participants having one of more tooth surfaces with incipient or overt caries at follow-up [29]. ICDAS 1 and 2 are initial non-cavitated lesions and it is possible that the predictors for these incipient lesions could be different from those for advanced caries, viz: scores from ICDAS 3 to 6.

### Explanatory variables

Information on all explanatory variables was collected at baseline. Saliva flow rate, buffering capacity and pH were recorded before the oral examination. Participants were instructed not to smoke, consume food or drink, brush the teeth or use a mouth wash for at least one hour prior to the scheduled appointment time: classes were rostered to attend the examination venue sequentially between 0900 and 1300 h. A sample of stimulated saliva (by chewing on a piece of wax) was collected over 5 min and used to measure the variables with GC SalivaCheck test kits (http://www.gcaustralasia.com/Products/97/Prevention/Saliva-Check-BUFFER). Saliva was expectorated into a collection cup and the volume per 5 min calculated. The litmus strip from the test kit was dipped into the collection cup and pH recorded after 30 s. Buffering capacity, defined as the amount of acid or base that can be added without changing the pH by more than 1 pH unit, was recorded on the colorimetric strips provided. Caries Risk Test (CRT) agar plates were used to quantify cariogenic bacteria and yeast colonies (http://www.ivoclarvivadent.com/en/p/all/products/prevention-care/caries-risk/crt-bacteria). Agar plates were coated with saliva and incubated for 48 h at 37 °C according to manufacturer guidelines. Colony forming units (CFU) were determined by referring to CRT guideline charts. For MS and LB this was recorded as < 10^5^ CFU/ml saliva or ≥ 10^5^ CFU/ml: saliva and Yeast counts were categorized into either none/light or moderate/heavy. In addition, sex, being in either the intervention or the comparison group and baseline caries experience (caries experience from ICDAS 1–6) were taken as explanatory variables.

### Statistical analyses

SPSS 26.0 (IBM, New York) was used. Descriptive statistics were carried out to estimate the frequencies and means, as required. Inter-examiner reliability for ICDAS was tested using Kappa statistics, where 5% of children were re-examined by the three participating examiners. The overall Kappa was 0.837, indicating a high level of agreement. Sex, being in either the intervention or comparison group, presence or absence of caries at baseline, baseline salivary pH (≤ 6.6 and > 6.6), flow rate (≤ 5 ml/5 min and > 5 ml/5 min), buffering capacity (≤ 9 and > 9), salivary loads of MS and LB (> 10^5^ and ≤ 10^5^ CFU/ml saliva) and yeast (moderate or heavy v none or light) counts were dichotomised for descriptive analysis and presented as numbers and percentages. Chi Square tests were performed to compare caries incidence in relation to explanatory variables. We also used Generalised Linear Models with negative binomial regression and log link to explore the association between the explanatory variables and the outcomes. At first, a univariate analysis was conducted to assess the effect of each explanatory variable on the outcome variable, by entering one variable at a time. A multivariable analysis was then conducted to explore the association between explanatory variables where all variables were entered at once. Exponential estimates of the negative binomial regression with log link analyses are presented as Incidence Rate Ratios (IRR) with 95% confidence intervals (95% CI). For univariate and multivariable negative binomial regression analyses, salivary pH, buffering capacity and flow rate were used as scale measurements and for all tests, a p-value of < 0.05 was considered statistically significant.

## Results

Out of a total 408 participants at baseline, only 208 were present: all of these are included in the current analysis. There were more female participants (n = 119): 91 children in the group which received the preventative intervention; 117 in the comparison group (Table 1).

**Table 1:** Characteristics of the participants (used in the analysis) at baseline

Variable	N (%)
*Sex*	
Male	85 (41.7)
Female	119 (58.3)
*Comparison versus experimental group*	
Experimental group	117 (56.2)
Comparison group	91 (43.8)
*Baseline caries*	
Without caries (ICDAS 0)	14 (6.7)
With caries (ICDAS 1–6)	194 (93.3)
*Salivary pH at baseline*	
< 6 (low)	29 (13.9)
≥ 6 (high)	179 (86.1)
*Salivary flow rate per 5 min at baseline*	
< 5 ml (low)	148 (71.2)
≥ 5 ml (high)	60 (28.8)
*Total buffering capacity at baseline*	
< 9 (low)	83 (39.9)
≥ 9 (high)	125 (60.1)
*Salivary MS levels at baseline*	
≥ 10^5 CFU/ml saliva (high risk)	102 (69.4)
< 10^5 CFU/ml saliva (low risk)	45 (30.6)
*Salivary LB levels at baseline*	
≥ 10^5 CFU/ml saliva (high risk)	52 (35.4)
< 10^5 CFU/ml saliva (low risk)	95 (64.6)
*Salivary yeast counts*	
Moderate or heavy	24 (16.7)
None or light	120 (83.3)

For some variables, the number of participants does not add up to the total sample, due to missing data

Most had carious tooth surfaces (ICDAS 1 to 6) at baseline (93%). Nearly 90% of children had salivary pH levels over 6 and ~ 60% had a high salivary buffering capacity at baseline (Table 1). Not all children had both deciduous and permanent teeth, hence the total number of children varied at analysis. The majority of the children showed high levels of MS, LB and yeast in their saliva. Table 2 presents the results from the Chi-square analyses with the caries incidence in relation to sex, group allocation, baseline caries status, salivary pH, flow rate, buffering capacity, and salivary bacteria and yeast levels.

**Table 2:** Caries increment at 2 years in relation to sex, baseline salivary characteristics and caries status

Variable	Incipient caries (ICDAS 1 to 2)			Advanced caries (ICDAS 3 to 6)		
	No caries at 2 year follow up (%)	With caries at 2 year follow up (%)	p	No caries at 2 year follow up (%)	With caries at 2 year follow up (%)	p
*Sex*			0.89			0.76
Male	12 (14.1)	73 (85.9)		39 (45.9)	46 (54.1)	
Female	16 (13.4)	103 (86.6)		52 (42.3)	71 (57.7)	
*Comparison versus experimental group*			**0.01**			**0.02**
Experimental group	22 (18.8)	95 (81.2)		61(52.1)	56 (47.9)	
Comparison group	6 (6.6)	85 (93.4)		33(36.3)	58 (63.7)	
*Baseline caries status*			**0.01**			**0.04**
Without caries(ICDAS 0)	5 (35.7)	9 (64.3)		10 (71.4)	4 (28.6)	
With caries (ICDAS 1–6)	23 (11.9)	171 (88.1)		84 (43.3)	110 (56.7)	
*Salivary pH at baseline*			0.52			0.45
≤ 6	5 (9.6)	24 (82.8)		15 (51.7)	14(48.3)	
> 6	23 (12.8)	156 (87.2)		79 (44.1)	100 (55.9)	
*Salivary flowrate per minute at baseline*			0.97			0.21
≤ 5 ml	20 (13.5)	128 (86.5)		71 (48.0)	77 (52.0)	
> 5 ml	8 (13.3)	52 (86.7)		23 (38.3)	37 (61.7)	
*Total buffering capacity at baseline*			0.37			0.89
≤ 9	9 (10.8)	74 (89.2)		38 (45.8)	45 (54.2)	
> 9	19 (15.2)	106 (84.8)		56 (44.8)	69 (55.2)	
*Salivary MS levels at baseline*			**0.04**			**0.01**
> 10^5^ CFU/ml saliva	10 (9.8)	92 (90.2)		42 (41.2)	60 (58.8)	
≤ 10^5^ CFU/ml saliva	10 (22.2)	35 (77.8)		29 (64.4)	16 (35.6)	
*Salivary LB levels at baseline*			0.97			0.70
> 10^5^ CFU/ml saliva	7 (13.5)	45 (86.5)		24 (46.2)	28 (53.8)	
≤ 10^5^ CFU/ml saliva	13 (13.7)	82 (86.3)		47 (49.5)	48 (50.5)	
*Salivary yeast counts*			0.23			0.55
Moderate or heavy	5 (20.8)	19 (79.2)		13 (54.2)	11 (45.8)	
None or light	15 (12.5)	105 (87.5)		57 (47.5)	63 (52.5)	

X^2^ Chi Square statistics, figures in bold are significant (p < 0.05). For some variables, number of participants does not add up to the total sample, due to missing data

There were significant differences between experimental and comparison groups with regards to caries increment at the 2-year follow-up. Fewer children in the experimental group demonstrated advanced caries (ICDAS 3–6) at the follow-up than in the comparison group (p = 0.02). Amongst those who were caries free at baseline (ICDAS 0), significantly fewer children presented with incipient (p = 0.01) and advanced (p = 0.04) caries after two years than those children who had caries at baseline. A greater proportion of children with high salivary loads of MS at baseline had new incipient (90%) and advanced (59%) caries at two years than their counterparts who had low salivary loads of MS (Table 2).

Univariate analysis (Table 3) revealed that children in the experimental group experienced fewer surfaces with advanced caries by a factor of 33% (IRR 0.67; CI 0.48–0.95) compared to children in the comparison group. Having caries experience (ICDAS 1–6) at baseline, increased advanced caries by 2% (IRR 1.02; CI 1.01–1.04). With each unit increase in salivary flow rate, advanced caries increment decreased by 57% (IRR 0.43; CI (0.27–0.65). Compared to their counterparts who had lower salivary loads of MS, children with higher loads of MS experienced more advanced carious surfaces by 66% (IRR 1.66; CI 1.04–2.65). Similarly, children with higher loads of salivary LB levels had more surfaces with advanced caries than those with low salivary LB loads (IRR 1.84; CI 1.20–2.80).

**Table 3:** Univariate analysis with caries increment at 2 years as the outcome variable and salivary characteristics as explanatory variables

Variable	Incipient caries (ICDAS 1 to 2)			Advanced caries (ICDAS 3 to 6)		
	B (SE)	IRR (95%CI)	p	B (SE)	IRR (95% CI)	p
*Sex*						
Male	0.11 (0.16)	1.12 (0.82–1.51)	0.47	0.09 (0.17)	1.09 (0.77–1.55)	0.61
Female	0^a^			0^a^		
*Comparison versus experimental; group*						
Experimental group	− 0.23 (0.15)	0.80 (0.59–1.07)	0.13	**− 0.38 (0.17)**	**0.67 (0.48–0.95)**	**0.02**
Comparison group	0^a^			0^a^		
Baseline caries status	0.01 (0.01)	1.01 (0.99–1.02)	0.31	**0.02 (0.01)**	**1.02 (1.01–1.04)**	**0.02**
Salivary pH at baseline	− 0.12 (0.20)	0.88 (0.60–1.30)	0.53	− 0.02 (0.22)	0.97 (0.63–1.50)	0.91
Salivary flowrate per minute at baseline	0.08 (0.18)	1.08 (0.76–1.53)	0.66	**− 0.86 (0.22)**	**0.43 (0.27–0.65)**	**0.000**
Total buffering capacity at baseline	− 0.05 (0.05)	0.95 (0.86–1.05)	0.35	0.06 (0.05)	1.06 (0.95–1.18)	0.27
*Salivary MS levels at baseline*						
≥ 10^5 CFU/ml saliva	**0.42 (0.19)**	**1.53 (1.03–2.24)**	**0.03**	**0.50 (0.23)**	**1.66 (1.04–2.65)**	**0.03**
< 10^5 CFU/ml saliva	0^a^			0^a^		
*Salivary LB levels at baseline*						
≥ 10^5 CFU/ml saliva	0.35 (0.18)	1.42 (0.99–2.05)	0.06	**0.61 (0.21)**	**1.84 (1.20–2.80)**	**0.004**
< 10^5 CFU/ml saliva	0^a^			0^a^		
*Salivary yeast counts*						
Moderate or heavy	0.03(0.24)	0.97 (0.60–1.56)	0.90	− 0.42(0.31)	0.65 (0.35–1.18)	0.16
None or light	0^a^			0^a^		

0^a^ Reference category, p values in bold are significant

In the fully adjusted multivariable model (Table 4), there was a 58% reduction (IRR 0.42; CI 0.25–0.71) in advanced caries among children with increased salivary flow rates. Caries increment in those with higher salivary MS counts was 61% (IRR 1.61; CI 1.04–2.50) more for incipient caries and 121% (IRR 2.21; CI 1.24–3.92) more for advanced caries than those with lower salivary loads of MS.

**Table 4:** Multivariable analysis with caries increment at 2 years as the outcome variable and salivary characteristics as explanatory variables

Variable	Incipient caries (ICDAS 1 to 2)			Advanced caries (ICDAS 3 to 6)		
	B (SE)	IRR (95%CI)	p	B (SE)	IRR (95%CI)	p
*Sex*						
Male	0.12 (0.20)	1.13 (0.75–1.68)	0.56	0.23 (0.25)	1.26 (0.77–2.08)	0.36
Female	0^a^			0^a^		
*Comparison versus experimental group*						
Experimental group	− 0.29 (0.20)	0.75 (0.51–1.12)	0.16	0.35 (0.25)	1.43 (0.89–2.31)	0.15
Comparison group	0^a^			0^a^		
Baseline caries status	− 0.007 (0.01)	0.99(0.97–1.01)	0.54	− 0.007 (0.01)	0.99 (0.97–1.02)	0.62
Salivary pH at baseline	− 0.07 (0.23)	0.93 (0.59–1.46)	0.76	− 0.044 (0.25)	0.96 (0.58–1.57)	0.86
Salivary flowrate per minute at baseline	− 0.04 (0.20)	0.96 (0.65–1.42)	0.85	− 0.870 (0.27)	**0.42 (0.25**–**0.71)**	**0.001**
Total buffering capacity at baseline	− 0.04 (0.05)	0.96 (0.87–1.07)	0.45	0.005 (0.07)	1.01 (0.88–1.14)	0.94
*Salivary MS levels at baseline*						
≥ 10^5^ CFU/ml saliva	**0.48 (0.22)**	**1.61 (1.04**–**2.50)**	**0.03**	**0.79 (0.29)**	**2.21 (1.24**–**3.92)**	**0.007**
< 10^5^ CFU/ml saliva	0^a^			0^a^		
*Salivary LB levels at baseline*						
≥ 10^5^ CFU/ml saliva	0.34(0.21)	1.40 (0.93–2.11)	0.11	0.453 (0.25)	1.57 (0.96–2.57)	0.07
< 10^5^ CFU/ml saliva	0^a^			0^a^		
*Salivary yeast counts*						
Moderate or heavy	0.02 (0.25)	1.02 (0.62–1.68)	0.94	− 0.351 (0.33)	0.70 (0.37–1.34)	0.28
None or light	0^a^			0^a^		

0^a^ Reference category, p values in bold are significant

## Discussion

In our study population, high salivary loads of MS were significantly associated with the increment of both incipient and advanced caries, after adjusting for the effect of other confounding variables. There is a dearth of literature which has explored the risk of caries increment in relation to oral microbial loads in Indigenous populations. Most studies have assessed socio‐economic, dietary, behavioural and family characteristics [30–33]. Nevertheless, we found that having ≥ 10^5^ CFU/ml salivary loads of MS was significantly associated with an increment of tooth surfaces with incipient and advanced caries after two years. High loads of salivary LB were also associated with advanced caries increment when compared to those with low salivary loads of LB. This is consistent with our baseline study where caries experience was approximately twice in those with higher counts of salivary MS than in those with lower counts [34], demonstrating that simple chair side assessment of MS would assist in determining the risk for future caries. This remains true even though we now recognise that a cariogenic/acidogenic microbiome can vary considerably and there is nothing highly specific about mutans Streptococci [35–38]. It is not surprising that our interventions designed to reduce total oral bacterial loads were found to be effective in preventing dental caries in this community [39]. Advanced investigations suggest that this could be due to a reduction of oral microbial diversity as a result of improved oral hygiene of the children [23]. As we learn more about oral microbiomics, it remains the case that *S. mutans* and closely related organisms of Genus Streptococcus can serve as marker species of a cariogenic microbiome, across human cultures.

Despite the fact that only our univariate analysis indicated a significant association between having caries at baseline and an increment in advanced caries, previous decay has been one of the most consistently demonstrated, significant predictors in caries risk assessment [40, 41]. This has been observed among Indigenous children in the US, Canada and Australia [22, 42], as well as amongst non-Indigenous children in these countries [40, 43]. Similar results were observed in recent studies in a population of low socio-economic African American children [44], in Brazil [45] and in Mexico [46].

Variations of saliva flow rate have been evaluated as a risk indicator for dental caries, especially among Caucasian populations [47], but there is a scarcity of such literature from Indigenous communities. Here, we have demonstrated a significant negative association between salivary flow rate and the increment of both incipient and advanced caries. Mean levels of salivary flow were reported to be lower in a population of Mexican adolescents with a caries score of DMFT ≥ 5 [48] and similar findings were reported in an Indian population [49] and among Ugandan adolescents [47].

Baseline salivary pH and buffering capacity were not associated with caries increment in the present work, possibly because these values vary throughout the day and a single reading is not representative.

When highest and lowest salivary loads were considered, the agreement between a chairside commercial test and real-time quantitative PCR for MS was nearly equal [50]. If participants followed the user instructions, the sensitivity and specificity of these chair-side caries risk assessment kits are high [51], and the results can be used to design targeted preventive interventions [52]. These simple risk assessment tools can be used in communities with limited resources. However, there are challenges in Indigenous communities due to limited access to dental services, and barriers to health services in general, which include individual, organizational and policy level determinants [53]. Even though chair-side tests are easy to use and quick [54], in rural underserved Indigenous communities, prerequisite equipment and resources may be unavailable [53, 55]. In addition, Indigenous Australians face many challenges and barriers in the utilization of oral health services, among them differences in perception of benefit within traditional health beliefs [53]. In this regard, ways in which Indigenous communities’ access public oral health services are important for execution: e.g. whether caries risk assessment should be done within mainstream or within Indigenous-specific clinics [56] or, as in our studies, in schools.

Limitations of this study include the high proportion of children who failed to attend one or more appointments with the research team. This is further discussed in other data papers from our study [39]. Many older children left the community for further education or employment as the study progressed. Families are frequently away for social or cultural reasons and truanting from schools is widespread. Unfortunately, it is a common experience for compliance to attend multiple oral examinations to be challenging in Indigenous communities [57].

For practical reasons saliva samples could not be taken at the same time of day from all participants, although most children participated during morning class hours. We cannot, therefore, account for diurnal variations in salivary physiology. We also have no control over what, if anything, children ate before attending: Pragmatism is essential in field studies of this type.

## Conclusion

Children who had reduced salivary flow rates and those with higher loads of salivary MS had a higher incipient and advanced caries increment after two years. As per unadjusted model, higher loads of salivary LB, low salivary flow rates, having a history of caries experience and being in the comparison group increased the risk of developing more surfaces with new advanced caries. We recommend that caries risk assessment and targeted preventive interventions be carried out in this and similar populations based on identified risk indicators. However, for effective and lasting impact, the many challenges faced by these communities related to wider public health access and service provision in the context of relevant and specific social and cultural factors need to be addressed with multidisciplinary interventions.

## Data Availability

All authors have full access to the all data (including statistical reports and tables) and gave final approval and agree to be accountable for all aspects of the work. Access to raw data may be requested from the corresponding author n.johnson@griffith.edu.au or from the Griffith University Human Research Ethics Committee (Ref: DOH/05/15/HREC) at research-ethics@griffith.edu.au.

## Abbreviations

NPA: Northern Peninsula Area
FNQ: Far North Queensland
ICDAS-II: International Caries Detection and Assessment system
CRT: Caries Risk Test
MS: Mutans streptococci
LB: Lactobacillus
CFU: Colony forming units
